# Supplementary material for: Senior Theater Projects: Enhancing Physical Health and Reducing Depression in Older Adults
Source: Int J Environ Res Public Health. 2024 Sep 26;21(10):1289. doi: 10.3390/ijerph21101289 (PMC11507429; doi:10.3390/ijerph21101289)
Supplement: Supplementary file 1 [file ijerph-21-01289-s001.zip › ijerph-3070336-supplementary.pdf]

Supplementary Table S1

Stage	Description
1 .warming-up	(Mind and Body Stretching Exercises)<br>Stretching of upper and lower extremities and trunk<br>Move your whole body freely to the music.<br>(Vocal training to improve smoothness)<br>Exercises for facial muscles(ex. Small squint of the eyes and mouth, then open wide and fully.)<br>Exercises for orbicularis oris muscle and tongue muscle<br>Vocalization by imitating animal noises
2. Emotional Release Training	(impromptu skit)<br>Expressing joy, anger, sorrow, and pleasure (ex. Express anger and pleasure you have felt recently)<br>Express the theme given by the instructor through body movements and facial expressions. (ex. Helping a person who has fallen, Confess to your first love)<br>Parrot back to the instructor's movements, facial expressions, and lines.
3. Theatrical practice	(First period)<br>Practice dance choreography<br>Think of your own lines for the scene.<br>Think about how you want your lines to be communicated.<br>Create props for use in the play<br>(Latter period)<br>memorize a script<br>Practice lines and acting together with more than one person.<br>Express yourself using your whole body to convey your emotions.
